# Predictors of Hospital Length of Stay among Patients with Low-risk Pulmonary Embolism

**DOI:** 10.36469/9744

**Published:** 2019-04-08

**Authors:** Li Wang, Onur Baser, Phil Wells, W. Frank Peacock, Craig I. Coleman, Gregory J. Fermann, Jeff Schein, Concetta Crivera

**Affiliations:** 1 STATinMED Research, Plano, TX, USA; 2 Department of Internal Medicine University of Michigan, Ann Arbor, MI; 3 Department of Medicine University of Ottawa and the Ottawa Hospital Research Institute, Ottawa, Ontario, Canada; 4 Emergency Medicine Baylor College of Medicine, Houston, TX; 5 School of Pharmacy University of Connecticut, Storrs, CT; 6 Department of Emergency Medicine University of Cincinnati, Cincinnati, OH; 7 Janssen Scientific Affairs, LLC, Titusville, NJ

**Keywords:** united states department of veterans affairs, comorbidity, burden of illness, pulmonary embolism, length of stay

## Abstract

**Background:** Increased hospital length of stay is an important cost driver in hospitalized low-risk pulmonary embolism (LRPE) patients, who benefit from abbreviated hospital stays. We sought to measure length-of-stay associated predictors among Veterans Health Administration LRPE patients.

**Methods:** Adult patients (aged ≥18 years) with ≥1 inpatient pulmonary embolism (PE) diagnosis (index date = discharge date) between 10/2011-06/2015 and continuous enrollment for ≥12 months pre- and 3 months post-index were included. PE patients with simplified Pulmonary Embolism Stratification Index score 0 were considered low risk; all others were considered high risk. LRPE patients were further stratified into short (≤2 days) and long length of stay cohorts. Logistic regression was used to identify predictors of length of stay among low-risk patients.

**Results:** Among 6746 patients, 1918 were low-risk (28.4%), of which 688 (35.9%) had short and 1230 (64.1%) had long length of stay. LRPE patients with computed tomography angiography (Odds ratio [OR]: 4.8, 95% Confidence interval [CI]: 3.82-5.97), lung ventilation/perfusion scan (OR: 3.8, 95% CI: 1.86-7.76), or venous Doppler ultrasound (OR: 1.4, 95% CI: 1.08-1.86) at baseline had an increased probability of short length of stay. Those with troponin I (OR: 0.7, 95% CI: 0.54-0.86) or natriuretic peptide testing (OR: 0.7, 95% CI: 0.57-0.90), or more comorbidities at baseline, were less likely to have short length of stay.

**Conclusion:** Understanding the predictors of length of stay can help providers deliver efficient treatment and improve patient outcomes which potentially reduces the length of stay, thereby reducing the overall burden in LRPE patients.

## Background

Pulmonary embolism (PE) is a potentially life-threatening medical emergency and the third major cause of cardiovascular death after myocardial infarction and cerebrovascular accidents.^1,2^ PE has an estimated incidence rate of 112 cases per 100 000 in the general population and is attributed to approximately 100 000 deaths per year.^1,3,4^ Additionally, risk of PE recurrence is exceptionally high, with 25% of recurrences within 5 years post-initial hospitalization.^5^

PE is associated with a substantial burden of health care utilization and associated costs.^5^ In the US, annual PE costs are estimated at $8.5 to $19.8 billion.^6^ PE-related initial hospitalization costs are estimated at $13 300 to $31 000 annually.^5^ Increased hospital length of stay (LOS) increases the clinical burden of the disease and adds to the already higher health care costs among hospitalized PE patients.^7^ Low-risk PE (LRPE) patients may be treated safely in an outpatient setting obviating a hospital stay.^5^

Several factors influence hospital LOS in PE patients, including patient demographic characteristics, physician resistance, medication security, difficulty in risk stratification, and lack of a uniform approach to risk stratification.^8^ Treatment of PE with warfarin increases the LOS, as it requires frequent monitoring.^9^ Estimates suggest that up to 50% of PE patients can be treated safely in an outpatient setting.^10^ LRPE patients can be identified using risk-stratification algorithms including the Geneva score, the Pulmonary Embolism Severity Index (PESI) score, the simplified PESI score, the In-hospital Mortality for PE using Claims data criteria, the Spanish score, the Davies criteria, the Home management exclusion criteria, and the Hestia criteria.^11^ Further, LOS reduction can reduce complications associated with longer hospital stay, thereby reducing health care costs in LRPE patients.^8,10^ Therefore, our main objective was to identify predictors of LOS in LRPE patients using real-world data.

## Materials and Methods

### Data Source

This longitudinal, retrospective cohort study utilized data from the Veterans Health Administration (VHA) during October 1, 2010 to September 30, 2015 (study period). The VHA is the largest integrated health care system in the US, providing care at 1245 health care facilities.^12^ The Medical Statistical Analysis Software Administrative Database provided electronic health data including patients’ medical, pharmacy, laboratory, and VHA health plan enrollment information.^13,14^ Inpatient/outpatient diagnoses as well as related laboratory and pharmacy claims were identified using International Classification of Diseases, 9th Revision, Clinical Modification (ICD-9-CM) codes and procedures (recorded using ICD procedure and Current Procedural Terminology codes).^13^ This retrospective database analysis did not involve the collection, use, or transmittal of individual identifiable data. As such, Institutional Review Board (IRB) approval to conduct this study was not required and considered exempt according to 45CFR46.101(b)(4): Existing Data & Specimens - No Identifiers. Both the data set itself and the security of the offices where the data are housed meet the requirements of the Health Insurance Portability and Accountability Act (HIPAA) of 1996. As data were deidentified, patient consent was also not required.

### Study Population

Included patients were aged ≥18 years, had ≥1 inpatient diagnosis for PE (ICD-9-CM codes 415.1, 415.11, and 415.19) during the identification period (October 1, 2011-June 30, 2015), had a prescription claim for an anticoagulant (unfractionated heparin, low-molecular-weight heparin, warfarin, or novel oral anticoagulants) during the index hospitalization, and had continuous health plan enrollment with medical and pharmacy benefits for ≥12 months prior to index hospitalization discharge (including hospital stay [baseline period] until 3 months post-index date or until death [follow-up period], whichever occurred first). The first inpatient claim date for PE diagnosis during the identification period was considered the initial diagnosis date; the discharge date was designated as the index date. Patients administered subcutaneous heparin during the hospital stay were not included, since many are given subcutaneous heparin as a prophylaxis for PE. Patients with a PE claim or any anticoagulant claim prior to index hospitalization were excluded.

Eligible PE patients were stratified using the simplified PESI criteria into LRPE and high-risk PE. The simplified PESI is a version of the PESI in which selected variables of the original score are included (age, history of cancer, history of chronic cardiopulmonary disease, pulse, systolic blood pressure, and oxygen saturation levels). Patients scoring 0 points are considered at low risk, and all others are considered at high risk. LRPE patients were further stratified into short LOS (≤2 days) and long LOS (>2 days) cohorts based on index hospital LOS.

### Baseline Measures

Patient demographics including age, gender, race, and body mass index were assessed during the baseline period. In addition, clinical characteristics including Charlson comorbidity index (CCI) score,^15^ past medical history (hospitalized deep venous thrombosis [ICD-9-CM codes: 451.1, 453], left ventricular dysfunction [ICD-9-CM code 429.9], and cardiac dysrhythmia [ICD-9-CM codes 427.0-427.9]), and the administration of various diagnostic tests were recorded. Patients with various clinical markers including troponin I/T, and natriuretic peptide testing results during index hospitalization were assessed.

### Outcome Measures

Predictors of index hospital LOS (short versus long) among LRPE patients were identified. Hospital LOS was considered the binary outcome variable. Patient characteristics such as gender, race, body mass index, CCI score, past medical history, diagnostic tests during the baseline period; clinical markers during the index hospitalization were included as covariates.

### Statistical Analysis

Descriptive statistics were provided for all study variables, including baseline demographic and clinical characteristics among the short and long LOS cohorts. Chi-square tests were used to evaluate the statistical significance of differences for categorical variables. Student t-tests were used to assess differences in the means of continuous variables. A point estimate (mean difference for continuous variables and relative risk for categorical variables) and 95% confidence intervals (CIs) were presented. Logistic regression was performed to identify the predictors of hospital LOS (short versus long) among LRPE patients. Covariates adjusted in the model included gender, race, body mass index, CCI score, past medical history, diagnostic tests during the baseline period, and clinical markers during the index hospitalization. Adjusted odds ratios (ORs) and 95% CIs were presented. All analyses were conducted using Statistical Analysis Software (Version 9.3).

## Results

### Characteristics of Study Subjects

**Figure 1.**
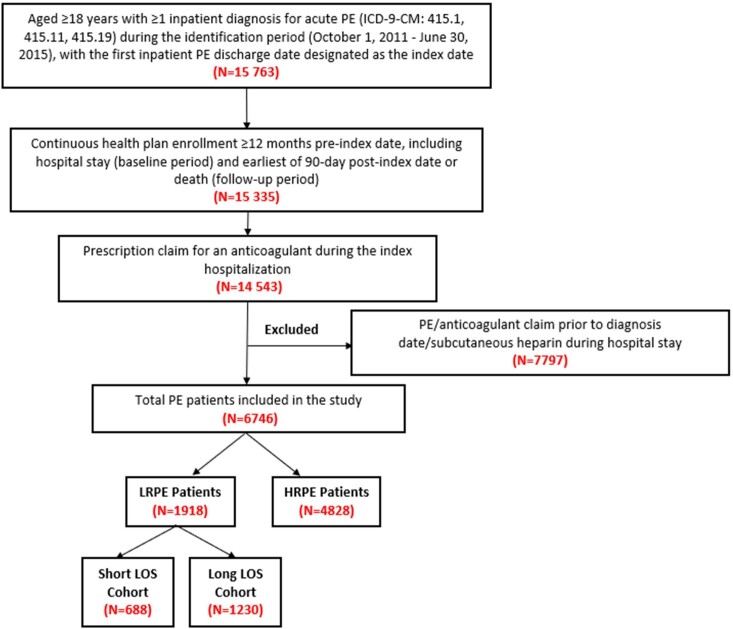
Patient Selection Criteria HRPE: high-risk pulmonary embolism; LOS: length of stay; LRPE: low-risk pulmonary embolism; PE: pulmonary embolism

After applying the inclusion and exclusion criteria, 6746 PE patients were included. Among these patients, 1918 (28.4%) met the definition of being LRPE patients, of whom 688 (35.9%) had a short index LOS and 1230 (64.1%) had a long index LOS (Figure 1). The average inpatient LOS during the index hospital stay was ~13 days and ~2 days in the long and short LOS cohorts, respectively. Additionally, the average length of follow-up was about 725 days and 746 days in the long and short LOS cohorts, respectively (data not shown).

### Baseline Characteristics

LRPE patients with a long LOS were older (60.7 vs 58.4 years, mean difference: 2.3, 95% CI: 1.28-3.37) and more likely to be male (94.6% vs 91.3%, relative risk: 1.04, 95% CI: 1.01-1.06) as compared to LRPE patients with a short LOS. Long LOS patients had higher CCI scores (1.1 vs 0.8, mean difference: 0.3, 95% CI: 0.16-0.42) and a higher proportion of patients with moderate or severe renal disease (20.3% vs 14.2%, relative risk: 1.4, 95% CI: 1.04-1.96), diabetes (29.3% vs 22.5%, relative risk: 1.3, 95% CI: 1.10-1.53), and cardiac dysrhythmia (16.4% vs 10.0%, relative risk: 1.6, 95% CI: 1.27-2.12) as compared to short LOS patients. The long LOS cohort had a higher proportion of patients with troponin I testing (38.1% vs 30.1%, relative risk: 1.3, 95% CI: 1.11-1.45) during index hospitalization as compared to the short LOS cohort (Table 1).

**Table 1.** Baseline Demographic and Clinical Characteristics of LRPE Patients with Long versus Short LOS

	Long LOS		Short LOS		Point Estimate	95% Wald Confidence Limits	
	(>48 hrs) Cohort		(≤48 hrs) Cohort				
	N=(1230)		N=(688)				
	N/Mean	%/SD	N/Mean	%/SD	MD/RR		
**Age**							
Mean, SD	60.7	11.1	58.4	11.2	2.3	1.28	3.37
Median	63		61				
18-45	117	9.50%	100	14.50%	0.7	0.51	0.84
46-64	608	49.40%	346	50.30%	1	0.9	1.08
65+	505	41.10%	242	35.20%	1.2	1.03	1.32
**Sex**							
Male	1164	94.60%	628	91.30%	1.04	1.01	1.06
Female	66	5.40%	60	8.70%	0.6	0.44	0.86
**Race**							
White	786	63.90%	437	63.50%	1	0.94	1.08
Black	319	25.90%	187	27.20%	1	0.82	1.11
Unknown	94	7.60%	40	5.80%	1.3	0.92	1.88
Other	31	2.50%	24	3.50%	0.7	0.43	1.22
**Body Mass Index**							
Body Mass Index (kg/m2)	31.6	10.3	31.2	6.6	0.4	-0.49	1.21
**Past Medical History**							
Charlson Comorbidity Index Score	1.1	1.5	0.8	1.3	0.3	0.16	0.42
Myocardial Infarction	67	5.50%	33	4.80%	1.1	0.76	1.7
Congestive Heart Failure	0	0.00%	0	0.00%			
Peripheral Vascular Disease	74	6.00%	40	5.80%	1	0.71	1.5
Dementia	15	1.20%	1	0.20%	8.4	1.11	63.38
Cerebrovascular Disease	128	10.40%	45	6.50%	1.6	1.15	2.21
Chronic Pulmonary Disease	81	6.60%	49	7.10%	0.9	0.66	1.3
Rheumatologic Disease or Connective Tissue Disease	21	1.70%	7	1.00%	1.7	0.72	3.93
Peptic Ulcer Disease	29	2.40%	4	0.60%	4.1	1.43	11.49
Mild Liver Disease	16	1.30%	8	1.20%	1.1	0.48	2.6
Hemiplegia or Paraplegia	0	0.00%	0	0.00%			
Moderate or Severe Renal Disease	250	20.30%	98	14.20%	1.4	1.04	1.96
Diabetes	360	29.30%	155	22.50%	1.3	1.1	1.53
Any Tumor (Other Malignancy)	18	1.50%	4	0.60%	2.5	0.55	11.62
Moderate or Severe Liver Disease	24	2.00%	6	0.90%	2.2	0.48	10.51
Metastatic Solid Tumor	0	0.00%	0	0.00%			
Diabetes + Complications	178	14.50%	64	9.00%	1.6	1.05	2.3
AIDS	90	7.30%	42	6.10%	1.2	0.49	2.93
Cardiac Dysrhythmia	202	16.40%	69	10.00%	1.6	1.27	2.12
Left Ventricular Dysfunction	33	2.70%	6	0.90%	3.1	1.3	7.31
Hospitalized Deep Vein Thrombosis	416	33.80%	208	30.20%	1.1	0.97	1.28
**Baseline Diagnostic Tests**							
Computed Tomography Angiography	453	36.80%	496	72.10%	0.5	0.47	0.56
Echocardiogram	24	2.00%	13	1.90%	1	0.53	2.01
Lung Ventilation/Perfusion Scan	23	1.90%	18	2.60%	0.7	0.39	1.32
Venous Doppler Ultrasound	210	17.10%	172	25.00%	0.7	0.57	0.82
**Clinical Marker during Index Hospitalization**							
# Patients with Troponin I, N	469	38.10%	207	30.10%	1.3	1.11	1.45
# Patients with Troponin T, N	19	1.50%	14	2.00%	0.8	0.38	1.5
# Patients with Natriuretic Peptide Testing, N	453	36.80%	223	32.40%	1.1	1	1.29

AIDS: acquired immune deficiency syndrome; LOS: length of stay; LRPE: low-risk pulmonary embolism; MD: mean difference; RR: relative risk; SD: standard deviation

### Predictors of Short versus Long LOS among LRPE Patients

**Figure 2.**
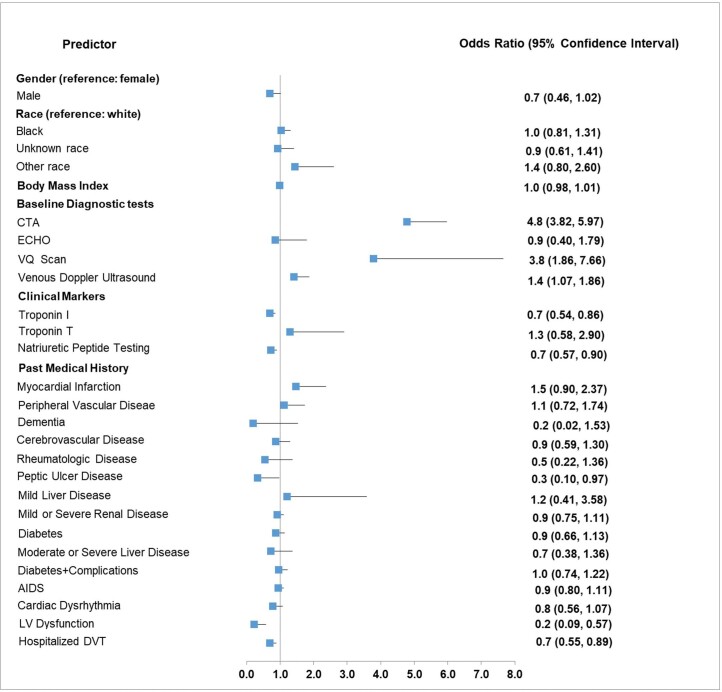
Predictors of Short versus Long LOS among LRPE Patients CTA: computed tomography angiography; DVT: deep vein thrombosis; ECHO: Echocardiogram; LOS: length of stay; LRPE: low-risk pulmonary embolism; LV: left ventricular; VQ: lung ventilation/perfusion

After adjusting for other covariates, LRPE patients with computed tomography angiography (OR: 4.8, 95% CI: 3.82-5.97), a lung ventilation/perfusion scan (OR: 3.8, 95% CI: 1.86-7.76), or venous Doppler ultrasound (OR: 1.4, 95% CI: 1.08-1.86) during the baseline period had increased probability of having a short LOS (Figure 2). Conversely, after adjusting for other covariates, LRPE patients with clinical markers troponin I (OR: 0.7, 95% CI: 0.54-0.86) or natriuretic peptide testing (OR: 0.7, 95% CI: 0.57-0.90) during index hospitalization; as well as those with past medical history of left ventricular dysfunction (OR: 0.2, 95% CI: 0.09-0.57), deep venous thrombosis hospitalization (OR:0.7, 95% CI: 0.55-0.89), or peptic ulcer disease (OR: 0.3, 95% CI: 0.10-0.97) during the baseline period; had a decreased probability of having a short LOS (Figure 2). Sex, race, and body mass index did not have any significant effect.

## Discussion

PE is one of the most important causes of hospitalization in the US.^16,17^ Hospital LOS is considered a potential indicator for the effectiveness of treatment in PE patients.^18^ In our study, we observed a negative association between LOS and diagnostic tests and a positive association between LOS and clinical markers and comorbidities. Our effort to identify predictors of hospital LOS in LRPE patients, in a real-world clinical and comorbidities. Our effort to identify predictors of hospital LOS in LRPE patients, in a real-world clinical setting, is unique and may have important clinical implications to aid clinicians in effective patient management. Study results showed that 28.4% of PE patients were classified as low risk. These results were consistent with Additionally, our study indicated that less than half (36%) of LRPE patients had a short LOS (≤2 days), despite being the recommendation for early discharge of LRPE patients.^19^ In a study by Shafiq et al, only 9% of PESI stratified LRPE patients were discharged early (≤3 days).^20^ According to the 2008 European Society of Cardiology guidelines, risk stratification of PE patients aids in identifying LRPE patients who can be managed separately with abbreviated hospital stays or in an outpatient setting.^21^

In our study, LRPE patients in the long LOS cohort were significantly older than those with short LOS. These results were consistent with a previous study by Berghaus et al, which showed that older age is independently associated with a longer hospital LOS.^17^ Our results showed that the short LOS cohort had a higher proportion of patients with diagnostic tests including computed tomography angiography and venous Doppler ultrasound in the baseline. Our study also showed that these diagnostic tests in the baseline are significantly associated with a shorter LOS, possibly due to physicians being extremely cautious in stratifying PE patients as low risk and performing more tests to confirm their early discharge/outpatient management. Previous studies showed that inappropriately selecting PE patients at higher risk for early discharge resulted in a higher post-discharge mortality.^17,22^ Therefore, early discharge/outpatient management should only be considered in LRPE patients who are accurately identified.

Our findings showed that the presence of clinical markers including troponin I and natriuretic peptides are significantly associated with a longer hospital LOS in LRPE patients. A meta-analysis conducted by Becattini et al showed that the increase in clinical markers troponin I/T was associated with severe medical complications and mortality in both the high-risk PE and hemodynamically stable LRPE patients.^23^ Medical complications might contribute to the longer LOS in these LRPE patients with clinical markers. Further, our results showed that individual comorbidities including left ventricular dysfunction, hospitalized deep venous thrombosis, and peptic ulcer disease are associated with a longer hospital LOS in LRPE patients. This is consistent with the findings of Berghaus et al evaluating the influence of LOS in PE patients on thrombolytic therapy, in which the presence of comorbidities including left ventricular dysfunction, renal disease, and cancer were independently associated with longer LOS.^17^ It is understandable that patients with comorbidities tend to have a longer LOS, as these comorbidities might increase the severity of the disease and prolong LOS to get them under control. In Donadini et al, the results of multivariate analysis showed that LOS was only associated with national early warning score, where PE patients with a score of ≥5 were more likely to have a longer LOS. National early warning score is a risk-stratification tool commonly used in clinical practice based on vital parameters: respiratory rate, oxygen saturation, temperature, systolic blood pressure, pulse rate, and level of consciousness.^24^ However, there are other factors associated with LOS in LRPE patients, including the anticoagulant chosen for the treatment of PE. Previous studies have showed that LRPE patients treated with rivaroxaban had a shorter LOS than those treated with standard vitamin K antagonists.^25^ Therefore, there is a need to conduct further research assessing the factors associated with LOS in LRPE patients.

Our findings should be viewed in the context of some study limitations. First, this study relied on retrospective claims data. While claims data are extremely valuable for the efficient and effective examination of health care outcomes, treatment patterns, and costs, they are collected for payment and not research. The presence of a diagnosis code on a medical claim is not a positive presence of disease and may be incorrectly coded or included as rule-out criteria rather than the actual disease. To ensure exclusion of any rule-out PE diagnoses, PE patients were required to have an anticoagulant claim during their hospital stay. The presence of a claim for a filled prescription does not indicate the medication was consumed or taken as prescribed. Prescriptions filled over the counter or provided as samples by the physician are not observed in claims data. Thus, the true number of medications prescribed may not be accurately recorded. Certain clinical and disease-specific parameters are not readily available in claims data, which could influence study outcomes. The results of our study are subject to bias and should be interpreted with caution, as the LOS stratification was based on the index hospital LOS, and the LOS predictors included the variables measured during the baseline period (12 months before the discharge date, including the index hospital stay) and the index hospitalization. Also, our study was only limited to those receiving treatment with anticoagulants, thus excluding patients that might have a contraindication to anticoagulants, who would potentially have a longer LOS. Additionally, the results should be interpreted with caution as the use of a different cut-off point to stratify the short and long LOS cohort might have led to different conclusions. The current study also represented only US data from a specific subpopulation (veterans), who were mostly elderly men. Therefore, the generalizability of our findings to young male patients or females requires further study.

## Conclusion

In summary, the results of our study showed that a majority of LRPE patients had a long LOS. Prior diagnostic tests were positive predictors, whereas clinical markers and individual comorbidities were negative predictors for a shorter LOS. However, more research is necessary to evaluate the association between LOS and diagnostic tests. Further research will aid in understanding the predictors of LOS to deliver efficient treatment and reduce the LOS for LRPE patients, which may reduce the overall burden of PE.

## Acknowledgements

Editorial assistance was provided by Michael Moriarty and Chris Haddlesey of STATinMED Research.

## Ethics Approval

This retrospective database analysis did not involve the collection, use, or transmittal of individual identifiable data. As such, Institutional Review Board (IRB) approval to conduct this study was not required and considered exempt according to 45CFR46.101(b)(4): Existing Data & Specimens - No Identifiers. Both the data set itself and the security of the offices where the data are housed meet the requirements of the Health Insurance Portability and Accountability Act (HIPAA) of 1996.

## Funding Statement

This study was funded by Janssen Scientific Affairs.

## Competing Interests

WFP has received grants from Abbott, Alere, Banyan, Cardiorentis, Janssen, Portola, Pfizer, Roche, and ZS Pharma; is a consultant to Alere, Beckman, Boehringer-Ingelheim, Cardiorentis, Instrument Labs, Janssen, Phillips, Portola, Prevencio, Singulex, The Medicine’s Company, and ZS Pharma; and also has ownership interests at the Comprehensive Research Associate LLC, Emergencies in Medicine LLC.

CIC has received grant funding and consulting fees from Janssen Scientific Affairs, LLC, Raritan, NJ and Bayer Pharma AG, Berlin, Germany.

PW receives speaker fees from Bayer Healthcare and Daiichi Sankyo, writing committee fees from Itreas, and grant support fees from Pfizer/BMS. [ORCID number 000-0002-8657-8326]

GJF has received research support from Novartis, Siemens, Pfizer, Portola, and PCORI; has advised Janssen Scientific Affairs, LLC; and receives speaker fees from Janssen.

CC and JS and are employees of Janssen Scientific Affairs.

LW is employee of STATinMED Research, which is a paid consultant to Janssen Scientific Affairs. [Bibr ref-9118]
